# Supplementary material for: Nonenveloped Avian Reoviruses Released with Small Extracellular Vesicles Are Highly Infectious
Source: Viruses. 2023 Jul 23;15(7):1610. doi: 10.3390/v15071610 (PMC10384003; doi:10.3390/v15071610)
Supplement: Supplementary file 1 [file viruses-15-01610-s001.zip › viruses-2477834-supplementary.pdf]

Supplementary Figure S1. The σC protein is present in EVs. The western blots were developed same as in Figure 3B, but analyzed by using chicken polyclonal antiserum against ARV. A very strong band was observed (as indicated by arrow in the left image in the EVs fractions, which was identified as σC by mass spectrometry. The band was excised from the right SDS-PAGE gel to analyze by mass spec. The dashed line is used to indicate the position of the band to compare the western blots with the gel stained by Gelcode™ Blue Stain Reagent.

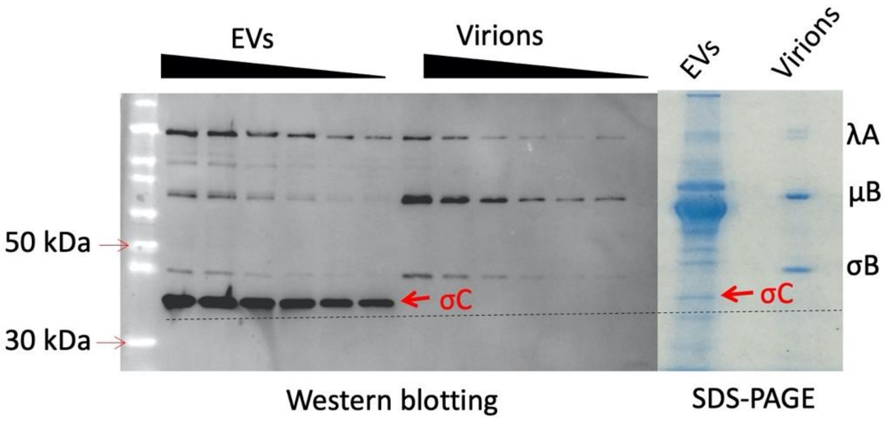

Supplementary Figure S2. Triton and sonication could not remove σC protein from the virions. The Western blots were the full image shown in the Figure 1C (top), which was probed with the chicken antibody against ARV virion.

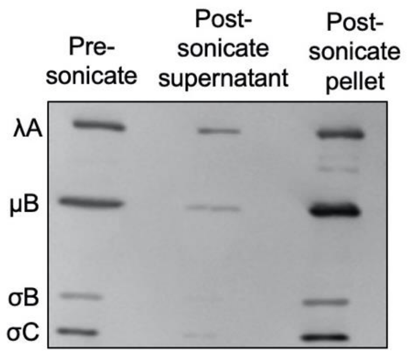

Supplementary Table S1. Mass spectrometry identifies the larger protein band as σC protein.

Unique Peptides	Peptides	Group Description	Molecular Weight (kDa)
3	14	σC	35.0
3	9	σC	
3	7	σC	
2	16	σC	
2	12	σC	
2	11	σC	
1	10	σC	
1	2	σC	
1	1	σC	
1	1	σC	
1	1	σC	
Total 20	Total 84		

Supplementary Video S1. The cryoEM tomogram of EVs.

exosome tomography movie.m4v
